# From enhancement to over-reliance: a mixed-method study of generative AI and sustainable learning performance

**DOI:** 10.3389/fpsyg.2026.1847369

**Published:** 2026-06-29

**Authors:** Li Gao, Yanli Sun, Safeer Ullah Khan

**Affiliations:** 1 Student Aid Management Center, Jiangxi Institute of Technology, Nanchang, Jiangxi, China; 2 Foreign Language College, Weifang University, Weifang, China; 3 College of Management, Shenzhen University, Shenzhen, China

**Keywords:** AI literacy, AI over-reliance, artificial intelligence, self-regulated learning, sustainable learning performance

## Abstract

The rapid integration of generative artificial intelligence (AI) in education has raised important questions regarding its impact on student learning outcomes. While prior research has primarily focused on AI adoption, limited attention has been given to its influence on sustainable learning performance and the potential risks associated with its use. Addressing this gap, this study develops a comprehensive model to examine both the positive and negative effects of AI use on learning by integrating AI literacy, self-regulated learning, cognitive offloading, and individual differences. A mixed-method approach was employed, combining time-lagged survey data collected in three waves from 623 university students in China with qualitative interviews conducted with educators. The quantitative data were analyzed using PLS-SEM, while qualitative data were examined through thematic analysis to provide deeper insights into the observed relationships. The findings reveal that AI literacy significantly enhances critical AI evaluation, which, along with self-regulated learning, promotes effective AI use. Effective AI use positively influences sustainable learning performance but also increases AI over-reliance, which negatively affects learning outcomes. Furthermore, polychronicity moderates key relationships, indicating that multitasking tendencies shape both AI dependency and learning effectiveness. The qualitative findings support and explain these results, highlighting the behavioral mechanisms underlying AI-supported learning. Overall, the study demonstrates that the impact of AI on learning is dual in nature and depends on how students engage with the technology, offering important implications for theory and educational practice.

## 1 Introduction

Higher education is currently experiencing an unprecedented amount of opportunities and challenges due to the rapid adoption of Generative Artificial Intelligence (AI) technologies (AlAli and Wardat, 2024). The introduction of AI-based applications and Generative AI platforms opens up new avenues for increased student comprehension as well as individualized support in the form of advice and guidance (Tariq, 2025). Recent research suggests that the use of AI may facilitate students' learning by helping them understand complex concepts, providing immediate feedback and assisting students in problem solving (Akram et al., 2025). However, utilizing AI in the most effective manner requires that students actively participate and engage with these tools. Therefore, investigating how AI affects student learning performance is an important area of research for many academics. However, it is important to note that the effect of generative AI on students' learning is not always positive, but dual in essence. While using AI technologies strategically, students have the opportunity to deepen their understanding and retain knowledge better, while, on the other hand, excessive AI usage might result in an impairment of cognitive processes necessary for independent learning. The problem of dependence on AI technology from a cognitive perspective needs further research and theoretical elaboration.

While prior studies concentrated on different aspects of AI, including to what extent users trust the technology, perceive it as valuable, and have ethical issues (Yu et al., 2025; Zhang et al., 2025; Chiu, 2025), they failed to consider the learning implications of using AI. Most literature that deals with AI usage within education focuses on technology acceptance models such as TAM and UTAUT that are mainly concerned with the intention to use and the perception of usefulness rather than learning results. In essence, the earlier research in this field leaves a vital gap because it fails to address how AI usage impacts sustainable learning and dependency problems that result from AI usage. Moreover, there is a lack of literature focusing on LLM-based generative AI applications in education; most studies are based on a general digital tool approach that fails to account for the specific strengths and weaknesses of generative AI. Cognitive ability, self-regulatory skills, and academic motivation interact to determine how much learners benefit from AI or how negatively it affects their learning. The importance of the research gap stems from the fact that those students who use AI without having any training or critical thinking skills could become too reliant on AI and consequently lose interest in learning and retaining the subject matter. As such, this study aims to assess both the benefits and limitations of AI for students' performance to gain a complete picture of AI's impact on education.

This study draws on three complementary theoretical perspectives that are not only do they complement each other, but they are also merged into the dual-pathway model. The AI literacy framework is one of the foundations of the cognitive processes, as it shows how the knowledge about the advantages and disadvantages of AI technologies affects the results of using these technologies. Self-Regulated Learning (SRL) Theory extends this by describing how metacognitive planning and monitoring determine whether students interact with an AI productively or passively. Two theories explain how a good learning environment is created by the conscious and active use of artificial intelligence in the learning process, leading to sustainable and effective outcomes. Cognitive Offloading Theory provides a counterpoint to the environmental impact of effective AI use in promoting sustainable learning while creating the opportunity for cognitive over-reliance on AI by explaining that throughout time, when an individual delegates cognitive processing to external cognitive tools, the internal cognitive effort needed to create an engaged deep learning experience is reduced creating sustainable conditions for the habitual (over-) reliance on AI. The central theoretical contribution of this research is the recognition of the coexistence of two parallel processes in the creation of good learning environment through AI usage creating sustainable learning opportunities as well as creating cognitive over-dependency on AI use which is fundamentally different from prior models of cognition and AI adoption. AI literacy is a vital element in understanding how to best utilize artificial intelligence (AI); this refers to students' understanding of both what the capabilities are of an AI system (Chiu et al., 2024). AI literacy allows students to evaluate critically the outputs generated by AI(s), identify erroneous outputs, and make educated decisions on whether or not to use AI (Ng et al., 2024). Other characteristics of a high level of AI literacy are that these students will likely engage more in the process of critically evaluating any output generated by an AI system prior to verification, as well as meaningfully incorporating those outputs into their overall learning process (Göhler, 2025; Laupichler et al., 2025). Another aspect to consider regarding effective use of AI is self-regulated learning (SRL); this is the student's ability to regulate his/her own learning through planning, monitoring, and controlling his/her own learning process, to include strategies such as goal setting, time management, and utilization of various sources of learning. For example, in the case of AI, if a student has developed effective SRL skills, he/she is very likely to utilize AI tools in their study sessions in a way that will support the enhancement of his/her problem-solving ability and understanding of content material, rather than simply as an easy method of completing assigned work (Lima et al., 2026). In addition, the effective use of AI tools has the potential for additional risks; cognitive offloading theory suggests that over-reliance on an external tool may reduce one's cognitive efforts, thus diminishing one's capacity for critical thought and problem-solving (Gerlich, 2025). This challenge is even more pronounced in students with high levels of polychronicity (the tendency to multitask). Students with high levels of polychronicity may use an AI system whilst simultaneously engaged in other activities. This has the potential to decrease attention and therefore lessen the positive effects of AI on the learning process. The increased use of AI will thus exhibit a stronger relationship to the overall learning performance of students with lower levels of polychronicity and to a greater likelihood of over-reliance on AI by learners with greater levels of polychronicity.

Given these considerations, this study addresses the following research questions

***RQ1:*** How do AI literacy and self-regulated learning influence students' effective use of generative AI for learning?
***RQ2:*** How do effective AI use and AI over-reliance impact sustainable learning performance?
***RQ3:*** Does polychronicity moderate the relationships between effective AI use, AI over-reliance, and sustainable learning performance?

The current research makes four different contributions to the literature. Firstly, it goes beyond the technology acceptance model by focusing on the actual learning outcomes of the application of generative AI, measured through sustainable learning performance, which is the extent to which students perceive themselves as able to retain knowledge, learn independently, and transfer knowledge across contexts over time. This construct explicitly extends beyond the immediacy of learning outcomes in order to address the persistence and transferability of learning facilitated or impeded by AI engagement. Secondly, it develops a dual pathway model based on the theoretical explanation of paradox by way of applying SRL, AI literacy, and cognitive offloading theories to investigate how AI engagement contributes to the activation of both positive and risk pathways for sustainable learning. Thirdly, it introduces the concept of polychronicity as an individual difference variable, highlighting the effects of multitasking behavior on both opportunities and challenges presented by AI engagement in ways that have not been explored in previous literature. Finally, by incorporating a mixed methods approach in which the results of qualitative research in the form of educator interviews are integrated with PLS-SEM results through joint display analysis and meta-inference, a level of insight unattainable by quantitative studies alone is provided.

## 2 Theoretical background

### 2.1 Self-regulated learning (SRL) theory

Based on the SRL theory, the possibility of becoming a proactive learner lies in the capabilities of planning, monitoring, and regulating students' cognitive, motivational, and behavioral processes (Schunk and Zimmerman, 2003). Since the implementation of SRL allows defining learning goals, selecting the most appropriate strategies, managing time, and assessing progress, it is regarded by many teachers as the most important factor influencing learning success (Luo and Zhou, 2024). When it comes to using AI in a learning setting, the role of SRL becomes crucial because it implies that the student needs to learn how to employ different digital technologies, analyze AI-produced data (validate the outcome), and integrate them within the learning experience. In case learners lack the skills and capabilities of being self-regulated, they can easily mislead themselves and treat AI tools as shortcuts instead, thus affecting engagement and learning outcomes. Learners who have been successful at regulating their actions thanks to SRL will have no problems utilizing AI technology, e.g. use such solutions to grasp some particular concept, generate test questions, or receive adaptive feedback based on the personal preferences of the learner (Jin et al., 2023). The efficiency of self-regulation allows the learners to assess themselves, critically judge the AI outcomes, and adjust their learning process. Therefore, self-regulated learning emerges as an important feature in making the outcomes sustainable. Under the current framework, self-regulated learning will be the metacognitive mechanism through which students interpret how artificial intelligence can be used to facilitate their educational experiences and whether artificial intelligence serves as a tool for completing tasks efficiently or if artificial intelligence is an educational instrument. The primary difference between self-regulated learning and artificial intelligence literacy is that self-regulated learning allows for students to manage their own cognitive engagement, regardless of the technology they are using, while artificial intelligence literacy is limited to students' understanding of the various systems available for employing artificial intelligence in their education.

From the perspective of Chinese higher education, the SRL styles can be influenced by cultural and institutional factors that are not universally applicable. In collectivist learning settings, students are likely to feel social pressure from both their peers and educators, which affects goal-setting behaviors and monitoring in ways that are not similar to individualistic settings (Cortina et al., 2017). For example, in collectivist societies, a learner might feel compelled to finish his or her assignments early in order to meet his or her social responsibilities, thus interacting/engaging with artificial intelligence differently than individualistic learners. These cultural differences need to be understood when discussing SRL and the role of AI supported environments in determining effective use of AI. Within this particular research, SRL has been conceptualized as the antecedent of successful use of AI, representing the extent to which students metacognitively plan, monitor, and adjust their interaction with AI technologies for autonomous and meaningful learning.

### 2.2 AI literacy framework

AI literacy refers to the ability to understand, interpret, and critically evaluate AI systems, including their capabilities, limitations, and potential implications (Long and Magerko, 2020). It's not just having knowledge of what AI can do technically, but also possessing cognitive and ethical awareness so that students can interact with AI in a responsible and effective manner (Biagini, 2025). AI Literacy is distinct from Digital Literacy because of the need for students to use critical thinking to assess what is produced by AI; by using critical thinking, students are able to confirm, analyze and use AI in their learning process rather than simply accepting whatever the information is from the AI. AI literacy is also theoretically distinct from Critical AI Evaluation, which is a behavioral outcome of AI literacy rather than a component of it. Specifically, AI literacy represents the knowledge and cognitive awareness that a student brings to an AI interaction, whereas Critical AI Evaluation refers to the active, situated behavior of verifying, questioning, and cross-referencing AI-generated outputs during a specific learning task. A student may possess high AI literacy yet fail to engage in critical evaluation in a given instance due to time pressure, task complexity, or habitual reliance. This distinction justifies treating them as separate but causally linked constructs within the proposed model (Polomoshnov et al., 2025). Students with high levels of AI Literacy are able to incorporate critical evaluation of AI Output into the process of using AI effectively (Ng et al., 2024). As an example of this, a student with high AI Literacy may check the explanation created by AI against their textbook, identify any potential biases or inaccuracies associated with the explanation created by AI, and use the AI for support of their own learning rather than to eliminate the cognitive efforts being made. In contrast to this, students with lower levels of AI Literacy may tend to over-rely on AI for their learning, which will lead to less engagement with the material, a decreased ability to solve problems, and ultimately to lower levels of sustainable academic performance.

The inclusion of AI literacy within this framework supports the operationalization of sustainable learning performance as the primary outcome of interest. In this study, sustainable learning performance is defined as students' perceived capacity to retain knowledge over time, apply learning independently without AI assistance, and transfer understanding to new and different academic contexts. This definition is intentional in differentiating sustainable learning performance from instantaneous academic performance or efficiency in task completion, which means that it focuses on students developing sustainable, transferable skills and not instant outputs. The ability to be literate about AI helps students learn sustainably by allowing them to interact with AI-created material selectively and critically, hence maintaining cognitive involvement and knowledge retention. Students with a level of understanding of the limitations of AI are able to apply AI as a tool to improve comprehension, create an independent approach to solving problems, and improve long-term retention of knowledge (Du et al., 2025). At this time, AI is being rapidly integrated into higher educational systems in China (Wang et al., 2025), thus, AI literacy should be considered important since students are constantly overloaded with an immense academic load and resort to using AI as a convenient too (Sun et al., 2024). By having insufficient AI literacy, they are likely to overlook any quality assessment that may be available with the output of AI and therefore will become more dependent upon AI outputs. The incorporation of AI literacy in the framework creates room for understanding the positive impact of AI on learning and mitigating any threats associated with AI dependence. Importantly, the framework reinforces that the manner in which students engage with AI, rather than simply having access to AI tools, will ultimately determine whether they achieve sustainable learning outcomes. In the Chinese higher education setting, where students always have a lot of academic work and easy access to AI technologies, AI literacy acts as a key protective measure against AI dependence.

### 2.3 Cognitive offloading theory

The Cognitive Offloading Theory states that people are likely to use external tools to offload cognitive tasks because they have limited attention and must optimize the efficiency of their cognitive processes (Risko and Gilbert, 2016). Offloading may increase cognitive performance (e.g., completing tasks) and may, as a result, lower cognitive load; however, this practice could unintentionally cause people to become less engaged in deeper cognitive tasks, including problem-solving and critical thinking. Under the conditions of AI-assisted education, students may become too reliant on generative AI for the purpose of producing solutions to homework (Puvvadi et al., 2025). Based on the Cognitive Offloading Theory, the potential for an excess of reliance on AI would be considered a negative mediator because even students who demonstrate an understanding of how to use AI and possess self-regulated learning skills may tend to overdo it when employing AI as a cognitive offloading tool. The possibility of excessive reliance on AI would be even greater for students who exhibit a high level of polychronicity, a preference for multitasking. A polychronic student may easily shift attention from AI to their coursework or other tasks causing cognitive fragmentation, which may ultimately lead to decreased cognitive effectiveness and ultimately a lack of sustainable learning performance (Luo et al., 2023). Hence, the Cognitive Offloading Theory also serves as a theoretical basis for two different paths in the current conceptualization: the positive path, where the strategic offloading of cognitive tasks to AI creates opportunities for engaging in high-level cognition, and the negative path, where the habitual offloading of tasks without direction decreases cognitive activity internally and creates dependency. Within this framework, AI over-reliance is operationalized as a distinct construct that is conceptually related to but empirically separable from cognitive offloading *per se*. Consequently, AI over-reliance can be defined as a level at which students consistently and excessively rely on AI tools to perform academic tasks that they would be able to accomplish independently. Thus, AI over-reliance is distinguished from cognitive offloading, which is a neutral process of cognitive function transferring to different tools that may include many other tool types apart from generative AI tools, and from broader technology dependency, which concerns digital tool usage generally rather than generative AI learning consequences Specifically. In addition, it is different from automation dependency in human factors literature, where it usually applies to procedures or safety-critical areas as opposed to an educational setting. In defining AI over-reliance as a mediating variable that comes as a result of effective use of AI, this study captures a core paradox in that the same behaviors that constitute effective AI use can, under conditions of reduced self-regulation or elevated polychronicity, activate the over-reliance pathway and ultimately undermine sustainable learning performance.

## 3 Hypotheses development

### 3.1 AI literacy and critical AI evaluation

Students' knowledge and comprehension of AI systems—which includes knowing their capabilities, their limitations, and the possible errors of these systems—are reflected in their ability to think critically about AI systems (Long and Magerko, 2020). Higher AI literacy allows students to be better able to evaluate all types of AI responses to ensure the correctness of these responses and identify inconsistencies in them (Huang et al., 2023). Previous research has shown a strong correlation between the levels of digital literacy of students and the critical evaluation of digital information, allowing learners to differentiate between credible and non-credible digital information sources (Georgopoulou et al., 2025). In terms of of AI-assisted learning methods, the AI literate student is less likely to blindly accept the answers given by the AI system and more likely to engage in reflective assessment of those responses (Bewersdorff et al., 2025). AI Literacy Framework allows students to gain a foundational cognitive understanding of how an AI works; this foundation sets the framework for higher order cognitive evaluative processes. With the growing trend of AI implementation into higher education in China (Wu et al., 2020), students must possess sufficient AI Literacy to allow them to critically evaluate and/or assess the AI-informed suggestions made by the AI in order to minimize their exposure to false information, which negatively impacts independent learning. Unlike prior digital literacy research that examined evaluation of static web content, AI literacy in the context of generative AI requires students to assess probabilistic, dynamically generated outputs that may appear authoritative while containing errors or biases specific to large language model (LLM) behavior. This generative-AI-specific dimension of critical evaluation represents a theoretically distinct challenge not adequately captured by existing digital literacy frameworks, and provides a novel justification for the H1 pathway in the present model. Therefore, it is hypothesized that:

**H1:** AI Literacy positively influences Critical AI Evaluation.

### 3.2 Critical AI evaluation and effective AI use

Being able to critically evaluate AI outputs enables learners to strategically use AI tools in a way that encourages a deeper understanding of their learning rather than replacing the need for student cognitive efforts (Oates and Johnson, 2025). When students are able to reflect on the feedback given by the AI and confirm its correctness, they can integrate the AI into their study habits by using it to clarify a topic or as practice material for a particular problem (Dahlkemper et al., 2023). Research in educational technology demonstrates that learners who take the time to evaluate the digital resources that they are using are more likely to exhibit higher levels of engagement and have improved learning outcomes when compared with those learners who do not critically evaluate the resources they use (Nkomo et al., 2021). According to the Self-Regulated Learning Theory, whenever learners evaluate their performance metacognitively, they have better access to the tools and strategies needed for successful task completion and are more likely to monitor their progress toward achieving their goals (Schunk and Zimmerman, 2003). In AI-enabled learning environments, students who evaluate the accuracy of AI outputs are more competent at using AI to complement their learning by enhancing their ability to understand, retain, and develop skills (Melisa et al., 2025). In today's era, with an increasing number of students using AI in their coursework, critically evaluating the quality of AI outputs is crucial to ensure those students achieve substantial learning benefit from their AI usage (George, 2023). Critically, this relationship is theoretically distinct from general digital resource evaluation: generative AI outputs are produced interactively in response to student queries, meaning that the quality of critical evaluation directly shapes the AI interaction itself rather than simply filtering pre-existing content. This interactive dynamic makes the CAE-to-EAIU pathway specific to generative AI environments, providing novel justification extending beyond prior technology-enhanced learning models. Therefore, it is hypothesized that:

**H2:** Critical AI Evaluation positively influences Effective AI Use.

### 3.3 Self-regulated learning and effective AI use

SRL describes the student's ability to plan, monitor, and regulate their learning activities and achieve academic goals (Schunk and Zimmerman, 2003). The capability of the learner to demonstrate higher levels of SRL implies that the learner is able to set a clear goal; employ the right strategy for meeting the goal; and, after self-reflection and feedback, modify the strategy when necessary (Kizilcec et al., 2017). Because of the enhanced degree of SRL, learners who exhibit a high degree of SRL will also employ strategies involving AI in a strategic way (Xu et al., 2025). Earlier research has established that learners with a high level of SRL skills use digital learning resources more effectively, exhibiting higher levels of time on task and task completion rates (Anthonysamy et al., 2021). Metacognitive regulation, i.e., planning, monitoring, and self-reflection, is critical to effective learning with complex digital tools. Concerning the use of generative AI in particular, SRL becomes especially relevant due to the fact that AI applications generate products that may differ in terms of correctness and relevance based on the formulation of requests and the assessment of the generated response. Learners with high levels of SRL competence can benefit more from the interaction with generative AI by formulating learning-related objectives, monitoring whether the output of AI contributes to comprehension, and modifying their approaches to using AI when needed. This SRL-to-EAIU pathway thus captures a mechanism specific to AI-supported environments that goes beyond prior SRL research focused on traditional digital resources (Chang et al., 2023). When competing with high academic pressures and using multiple learning tools concurrently (Chen et al., 2025), SRL supports students in integrating AI into their learning purposefully, and helps students avoid excessive distractions or reliance on AI. SRL aids individuals to use AI with a goal-directed or reflective perspective, which ultimately leads to successful student outcomes and is therefore necessary for achieving sustainable learning outcomes (Zhang et al., 2025). Therefore, we propose:

**H3:** Self-Regulated Learning positively influences Effective AI Use

### 3.4 Effective AI use and sustainable learning performance

The effective integration of artificial intelligence into student learning engages the learner to be more involved with the material by providing opportunities to clarify complex concepts. This combined engagement creates a deeper understanding and retention of the knowledge gained through the use of AI in education (George, 2023). Studies have shown that students who make the effort to utilize AI for Study and Revision, tend to have higher Levels of Perceived Learning and to exhibit greater academic efficiency (Chen and Chang, 2024). Additionally, studies conducted on technological advancement's facilitation of improved cognition through Artificial Intelligence (AI) supporting and assisting learners who use technology has increased chronic impressions of memory (Yang, 2025). As a result, when evaluating the benefits of integrating Self-Regulated Learning (SRL) and Artificial Intelligence Literacy as theoretical frameworks, it is apparent why it would be beneficial for students to receive the assistance necessary with regard to their ability to reach long-term academic goals. The framework created by SRL states that students must develop plans, self-monitor their progress, and reflect on areas related to their learning process and apply them in conjunction with AI in order to gain a critical and productive understanding of the material being learned. Students who use AI as an active cognitive collaborator, rather than merely as a source of information, demonstrate increased depth of processing and greater independence in their ability to apply what they have learned. With respect to the example of China, where students are subject to high expectations of academic performance and numerous materials are accessible (Tian and Lu, 2017), properly integrating AI into learning environments will facilitate students' transition from merely consuming knowledge to producing knowledge and creating the foundational skill set necessary to maintain sustainable success. Although the case of China serves as the empirical setting, the underlying theory is believed to apply broadly in academic settings to cultural moderation. Therefore, it is hypothesized that:

**H4:** Effective AI Use positively influences Sustainable Learning Performance

### 3.5 Effective AI use and AI over-reliance

AI can be a valuable resource for learners, but it can also lead to an excessive dependency on AI where learners begin relying on AI for tasks that they would otherwise be capable of completing autonomously (Abubakar et al., 2025). Cognitive Offloading Theory explains how utilizing an external resource or tool to complete cognitive effort decreases the mental involvement of learners even if the resource or tool was intended to be used strategically (Risko and Gilbert, 2016). Research on students who regularly utilize an AI or digital learning tool has shown that these students exhibit habitual reliance on these tools and may under develop their independent learning ability and exhibit diminished critical thinking (Navas Bonilla et al., 2025). Moreover, there is some evidence that students with a high degree of literacy in using AI are still vulnerable to becoming excessively dependent on AI due to the ease with which it can be used to perform cognitive tasks that require high levels of cognitive effort, thus creating a paradoxical situation in which productive use of AI may inadvertently result in an over-reliance (Zhai et al., 2024). This paradox warrants deeper theoretical examination as it represents one of the most novel and counterintuitive contributions of the present model. This paradox occurs due to a particular cognitive habituation process, which implies that once students become accustomed to the efficiency of the AI, they will perceive the cost of engaging in cognition independently as higher than using AI, which makes the latter behavior a default option for solving cognitive problems. Cognitive offloading accounts for this process on the individual level, while habit formation research suggests that frequent, rewarding tool use bypasses deliberate metacognitive control over time. Consequently, students who use AI most effectively may be precisely those most exposed to habituation, since frequent productive engagement creates more opportunities for dependency to develop. The risk of over-reliance is further amplified in high-demand academic environments where cognitive resources are stretched (Kember, 2004). As such, it follows that effective AI usage will be positively correlated with AI dependence, which constitutes the key paradox to be explored in this study. Therefore, it is hypothesized that:

**H5:** Effective AI Use positively influences AI Over-Reliance

### 3.6 AI over-reliance and sustainable learning performance

Excessive reliance on artificial intelligence is associated with diminished student engagement and inhibited independent learning, with significant implications for sustainable learning outcomes (Elzerman, 2025). Research has shown that students who are dependent on AI to complete assignments tend to have lower levels of knowledge retention and substantially lower independent learning abilities, along with increased gaps in academic productivity (Karamuk, 2025; Zhang et al., 2024). Cognitive Offloading Theory states that by transferring cognitive processing outwards to AI applications, the amount of time spent on deep processing will be reduced and thus impede the ability to comprehend and transfer knowledge over a prolonged period of time (Gerlich, 2025). Even those who are proficient in using AI tools and monitor their usage may be prone to becoming overly dependent on them because of the ability for AI to reduce the complexity of tasks and reduce the effort required for cognitive engagement (Zhai et al., 2024). In the case of Chinese higher education, where students frequently face demanding academic workloads and widespread access to AI (Kember, 2004), dependency on AI impedes the development of critical habits of self-directed and self-regulated learning necessary to sustain knowledge application over time. When students habitually delegate cognitive effort to AI, the deep processing required for long-term knowledge consolidation is reduced, impairing the capacity to retain, transfer, and apply knowledge autonomously. The underlying cognitive mechanism is grounded in Cognitive Offloading Theory and is not culturally specific, though high-demand academic environments may amplify it. Therefore, it is hypothesized that:

**H6:** AI Over-Reliance negatively influences Sustainable Learning Performance

### 3.7 Moderating role of polychronicity

These three theoretical frameworks form a coherent rather than additive basis. SRL theory explains the cognitive regulation aspects underlying active interaction with technologies of AI. The AI literacy framework explains the knowledge regulation aspects underlying the critical assessment of AI-produced outputs. Finally, cognitive offloading explains potential dependency as a consequence of cognitive regulation activities being moved onto technologies of AI. Thus, these theories fit into the framework described above in the following way: AI literacy enables the ability to critically assess the information received (cognitive awareness), SRL enables the effective usage of AI (metacognitive regulation), effective use of technologies of AI creates opportunities for two ways to go – either toward achieving sustainable learning performance or becoming excessively dependent on these technologies (dual-pathway offloading), and polychronicity affects both pathways being an individual characteristic that regulates attention and cognitive engagement. This theoretical integration distinguishes this research from previous attempts to study AI in educational contexts where single-theory approaches and technology acceptance models, unable to capture learning quality as the desired outcome, have been used. Polychronicity is an individual tendency to accomplish several things at a time and frequently switch one's attention between these tasks (Howard and Cogswell, 2023). Studies have also shown that although multitasking appears to be efficient, there are signs of a reduction in attention, cognitive involvement, and learning retention not only in students utilizing digital technologies but also those effectively using traditional learning methods (Grawitch and Barber, 2013; Capdeferro et al., 2014). SRL Theory focuses on the fact that attention, goal-setting, and self-regulation are all essential aspects of effective learning using the aid of technology such as AI (Schunk and Zimmerman, 2003). Students with high polychronicity will find it difficult to maintain focus or regulate their learning activities in such a manner that will hinder effective use of AI technology in improving their sustainable performance during learning. Students who regularly engage in activities that divide their attention among multiple academic and non-academic responsibilities have been shown to experience fragmented cognitive processing and, as a result, have lower comprehension levels and less retention of knowledge (Glass and Kang, 2019). Within the context of Chinese higher education, the expectation that students manage large academic workloads along with many extracurricular activities makes it particularly appropriate to examine the role of high polychronicity in relation to engagement and achievement with AI. Thus, students with heightened polychronicity are expected to experience attenuated benefits from effective AI use, because fragmented cognitive processing reduces the depth of engagement needed to translate AI-assisted activity into durable learning. The moderating effect of polychronicity on the EAIU-SLP relationship therefore reflects an important individual-difference boundary condition on the positive pathway in the proposed model. Therefore, it is hypothesized that:

**H7a:** Polychronicity moderates the relationship between Effective AI Use and Sustainable Learning Performance, such that the relationship is weaker for students with higher polychronicity

Polychronicity refers to a person's preferred way of managing many activities at once and often shifting their focus from one task to another (Howard and Cogswell, 2023). While this might seem to help a person be more efficient, research on the topic reveals that engaging in many forms of multitasking may lead to a greater dependence on using outside resources to assist in managing their cognitive load and the completion of tasks within a shorter period of time (Himi et al., 2023). Cognitive offloading theory suggests that when a person's cognitive effort is taken over and distributed to resources outside themselves, the individual may establish a habitual dependency on those resources, even if the person has the ability to perform the task by themselves (Weis and Wiese, 2019). In the context of AI-supported learning, a student with high polychronic tendencies may have an increased tendency to rely on AI technology for handling a significant number of tasks concurrently, using it for cognitive laziness and developing stronger habits of relying on AI than those who are able to use AI to manage their learning efficiently. Studies have found that those who consistently multitask in their learning have an increased tendency to develop habits of overusing digital tools for assistance, reducing their ability to learn independently and engage critically with content (Wang et al., 2022). In China, where many students deal with extreme amounts of academic and outside commitments (Kember, 2004), having polychronicity will further develop a student's habitual overreliance upon AI-assisted learning resources. Importantly, this effect is theorized as an individual-difference moderator rather than a culturally specific phenomenon: the cognitive mechanism through which polychronicity amplifies AI over-reliance operates at the individual level and is not assumed to be exclusive to Chinese students. Therefore, polychronicity is expected to strengthen the association between effective AI use and AI over-reliance, reflecting a theoretically important boundary condition in the proposed dual-pathway model. Therefore, it is hypothesized that:

**H7b:** Polychronicity moderates the relationship between Effective AI Use and AI Over-Reliance, such that the relationship is stronger for students with higher polychronicity

**Figure 1:**
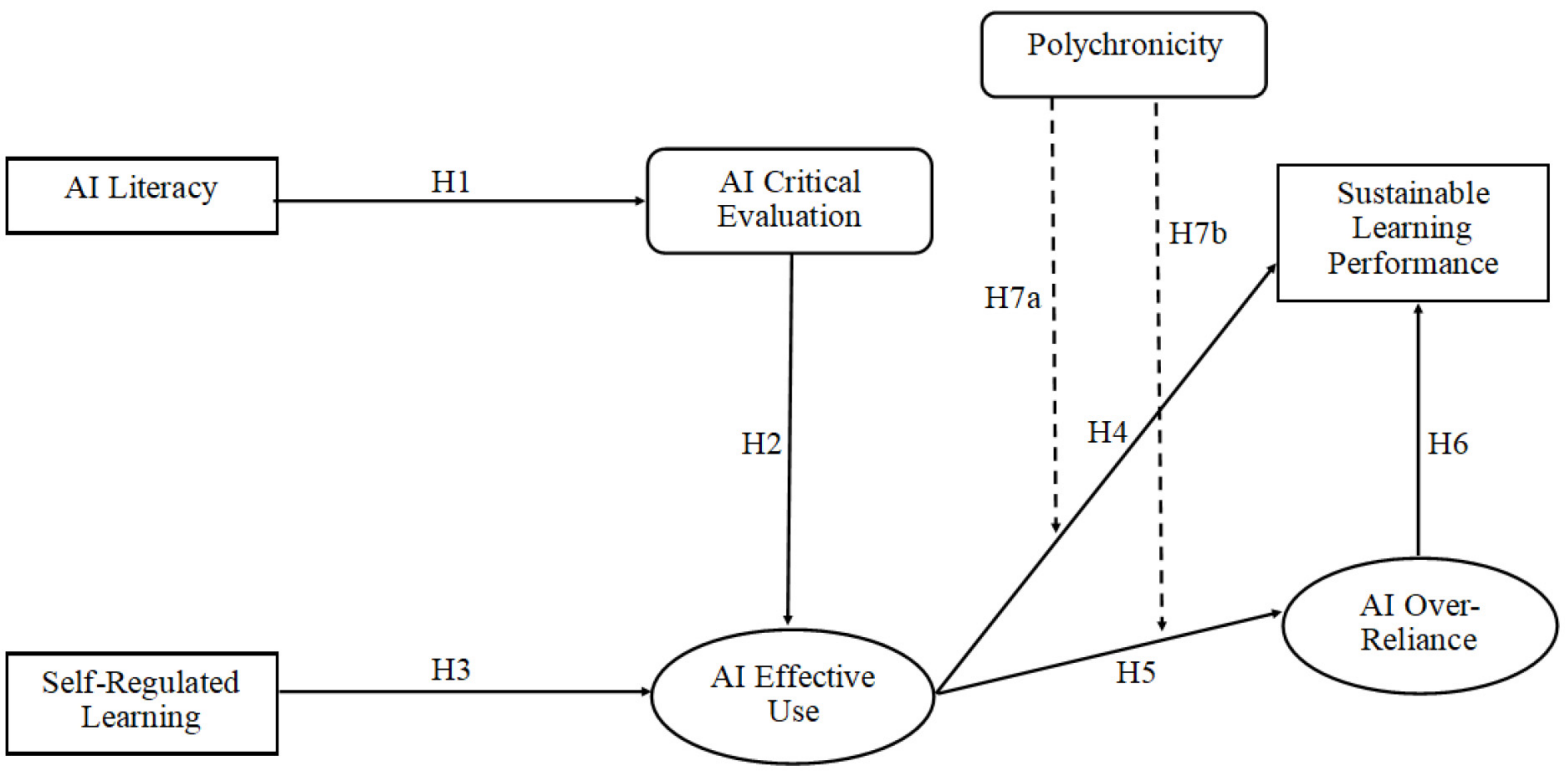
Conceptual model.

Figure 1 indicates the conceptual model.

## 4 Methodology

### 4.1 Data collection process

This study adopted an explanatory sequential mixed-method design, in which the quantitative phase was conducted first and the qualitative phase was subsequently used to explain, contextualize, and deepen understanding of the quantitative findings. This sequential structure reflects a deliberate integration strategy: the quantitative phase (PLS-SEM) was used to test and confirm structural relationships among constructs, while the qualitative phase (educator interviews) was conducted to generate explanatory insights into the mechanisms and contextual conditions underlying those relationships. Mixed-method designs of this type are recommended in educational and behavioral research precisely because they enable triangulation of findings, resolution of inconsistencies, and generation of meta-inferences that neither method could produce alone (Clark, 2019). Thus, in this investigation, while the qualitative phase (interviews) was conducted to provide explanations of contextual factors relating to the relationship between generative AI use by students and the effects on learning outcomes, the quantitative phase was used to test those established relationships between constructs within this study.

The objective of the quantitative part of the study was to collect information using a purposive sampling strategy directed toward Chinese university students who use generative AI tools while conducting their studies. Use of purposive sampling is advantageous when a participant needs to have knowledge or experience regarding a particular issue related to the research (Palinkas et al., 2015). The use of purposive sampling guarantees that the participants will be knowledgeable about AI tools, making the findings from their responses highly reliable and valid and making it appropriate for research in new and innovative fields such as artificial intelligence-supported learning. In addition, to provide greater methodological rigor and limit the possibility of bias in responses from participants, the present study employed a time-lagged design consisting of three waves of data collection over a 1-month interval. Time-lagged designs reduce the likelihood of common method variance (CMV) as well as the tendency for respondents to provide consistent or socially desirable responses (Podsakoff et al., 2003). Furthermore, creating temporal separation between the measurements used to collect data on a particular variable strengthens the inference of causality and limits the chance of reverse causality (Maxwell and Cole, 2007). Therefore, time lagged-designs are being recommended for use in behavioral research to enhance empirical findings.

The first wave involved gathering demographic information and independent variables such as self-regulated learning and artificial intelligence (AI) literacy from students attending Chinese universities. A total of 1,000 questionnaires were distributed resulting in 771 usable questionnaires returned to researchers resulting in 771 valid responses for a response rate of 77.1%. To facilitate follow-up participation for the second and third waves of the project, participants had the option to provide their email addresses or WeChat IDs, a common feature in longitudinal survey designs to maintain the same sample between periods of data collection (Dillman et al., 2014). Of the 771 respondents in the initial sample, 693 valid responses were collected for an approximate retention of 89.9% from Wave One. Wave Three, which occurred at the end of another month after Wave Two, included information regarding AI over-reliance, polychronicity, and performance regarding sustainability while learning. A total of 623 responses were obtained in the third wave, corresponding to a retention of 89.9% from Wave Two. The total matched sample of 623 participants resulted in an overall retention of approximately 62.3% across all three waves of data collection.

In order to ensure the quality of data collected in this study, all data was screened before analyzing them for completeness and consistency. Additionally, only fully matched responses across each wave of data collection were analyzed, which allows us to have confidence in the data's validity. The use of a time-lagged research design not only reduces the potential for common method variance (CMV) but also helps protect against problems associated with reverse causation and endogeneity (MacKenzie and Podsakoff, 2012). To further assess the potential for common method bias (CMB), two complementary statistical procedures were conducted beyond the time-lagged design, following established best-practice guidelines (Podsakoff et al., 2003; Kock, 2015). First, Harman's single-factor test was performed by entering all 47 items across the seven constructs into a single unrotated principal component analysis. The first unrotated factor explained only 37.22% of total variance, well below the 50% threshold, while the second factor accounted for an additional 15.21%, confirming a clearly multidimensional factor structure with no dominant common method factor. Second, the full collinearity variance inflation factor (VIF) procedure recommended by (Kock, 2015) was applied, in which each construct is regressed on all other constructs. All seven construct-level full collinearity VIF values were comfortably below the conservative threshold of 3.3: AI Literacy = 1.864, Critical AI Evaluation = 2.454, Self-Regulated Learning = 1.658, Effective AI Use = 3.236, AI Over-Reliance = 1.724, Sustainable Learning Performance = 1.655, and Polychronicity = 1.061. As a result, the use of both of the mentioned CMB diagnostics indicates that common method bias is not likely to constitute an important threat to the structural model's validity. The findings above, along with the three-wave time-lagged research design, indicate the presence of a high degree of methodological certainty concerning the validity of the results presented. However, ethical issues also needed to be taken into account during the research process. Firstly, respondents in the current research project were free to choose whether or not they would like to participate in the research and all the reasonable efforts have been made to ensure their anonymity and confidentiality. All the respondents received informed consent before they participated in the research and the respondents were aware of the fact that the data they provide will be used solely for research purposes, according to the existing ethical principles (Creswell et al., 2014). As a result, the data collection procedures used in the current study, which include purposive sampling and time-lagged design, form a credible and sound methodological framework for investigating the relationship between AI literacy, self-regulated learning, good utilization of AI, dependence on AI use, polychronicity, and learning effectiveness that could last for a long period of time. The demographics of respondents are shown in Table 1.

**Table 1:** Demographics profile (*n* = 623).

Demographic variable	Category	Frequency	Percentage
Gender	Male	207	33.20%
	Female	393	63.10%
	Other	23	3.70%
Academic level	Bachelor	198	31.80%
	Master	285	45.80%
	PhD	140	22.50%
Field of study	Engineering	120	19.30%
	Business	142	22.80%
	Computer Science	164	26.30%
	Social Science	125	20.10%
	Other	72	11.50%
AI experience	< 6 months	180	28.90%
	6–12 months	212	34.00%
	>1 year	231	37.10%

Data for the study were collected qualitatively, including semi-structured interviews, and conducted with a group of university educators and instructors from across China. Educators were targeted as a sample for this research since they have an extensive and varied background in observing students' learning behavior, as well as their use of AI tools and technology, along with the level of academic performance in AI-enabled learning environments. The researchers utilized purposive sampling to identify those educators that had experience working directly with students using generative AI tools for their academic work. The final sample of 15 educators was drawn from a variety of different academic disciplines, consistent with qualitative research guidelines, which suggest that a sample size of this nature is sufficient for establishing data saturation. All interviews were conducted online using a variety of platforms (e.g., Zoom, WeChat) based on the preferences of study participants, and each interview lasted approximately 30–45 min. Prior to conducting the interviews, all participants were briefed on the study and signed the informed consent document. All interviews were recorded and transcribed verbatim for analysis following each interview session. Thematic analysis was conducted following the six-phase framework of Braun and Clarke (2006): (1) familiarization with the data through repeated reading of transcripts; (2) generation of initial codes applied systematically across all transcripts; (3) collation of codes into candidate themes; (4) review of themes against the coded data and full dataset; (5) definition and naming of final themes; and (6) production of the thematic report. Two researchers independently coded a subset of transcripts to establish inter-rater reliability, and discrepancies were resolved through discussion. Data saturation was assessed iteratively; after the twelfth interview, no substantively new themes or categories emerged, confirming that the final sample of 15 educators was sufficient for thematic saturation consistent with qualitative research guidelines. Member checking was conducted with three participants to validate the accuracy and interpretive credibility of the identified themes.

### 4.2 Interview protocol

A semi-structured interview guide was developed based on the study constructs and hypotheses. The questions were designed to explore educators' perspectives on students' use of generative AI and its impact on learning outcomes.

Key themes included: The use of AI by students for academic and learning purposes & how effective AI is at enhancing students‘ ability to understand as well as perform academically; students' ability to critically reflect on and assess AI-generated content; too much reliance on AI is problematic; and how students can develop a self-regulatory approach when using AI. Examples of the types of questions asked include: (1) How do students generally utilize AI tools within the overall learning process? (2) Do you feel that the use of AI improves the way students comprehend subject matter, or do you believe it will lead to dependence on AI? (3) What methods do students utilize to assess and verify the accuracy of AI-generated material? (4) In your experience, have you been able to identify instances of students relying excessively on AI? (5) What role does the way that students choose to engage in multi-tasking behaviors (polychronicity) play in their use of AI tools?

### 4.3 Measures

All constructs used in this study were assessed with pre-existing validated scales that have been adapted from existing literature to validate their reliability and validity. Additionally, items on the questionnaire were adapted slightly to fit within the context of generative AI-supported learning experiences. A five-point Likert scale was utilized to determine level of agreement, where 1 = strongly disagree, and 5 = strongly agree, which has been widely utilized in the education and behavioral science fields (Hair et al., 2019).

The six-item scale developing AI literacy was based on the AI and Digital Literacy Frameworks (Yin et al., 2025), and focused on student's knowledge of AI's capability, functionality and limitations in terms of how these technologies work. Eleven items that were drawn from Multiple Self-Regulated Learning Scales were used to measure self-regulated learning (Alghamdi et al., 2020). A total of five items that had been derived from studies focused on critical reasoning and information evaluation in digital environments were used to measure the construct of Critical AI Evaluation (Lau et al., 2025). Effective use of AI was determined through nine items that had been previously developed from (Zhu and Yang, 2026). Over-reliance on AI was identified through five items adapted from several studies that had measured technology dependence (Acosta-Enriquez et al., 2025). This construct assesses the extent to which students excessively depend on AI for completing academic tasks. Polychronicity was measured using a five items scale adapted from (Franczak et al., 2024), which assesses individuals' preference for multitasking and handling multiple activities simultaneously. Sustainable Learning Performance was derived from a previous study (García-Morales et al., 2012) and reflects perceived long-term learning outcomes of the participant by using AI to retain and apply knowledge independently.

### 4.4 Data analysis

A combination of qualitative and quantitative methods was employed to analyze the data that has been collected. Quantitative analysis was conducted using Partial Least Squares Structural Equation Modeling (PLS-SEM) and the qualitative insights came from additional interviews with the educators. The quantitative component of the study initially examined the hypothesized relationships between Self-Regulated Learning, AI Literacy, Effective Use of AI, Critical Evaluation of AI, Over-Reliance on AI, Sustainable Learning Performance, and Polychronicity. For this purpose, SmartPLS 4 was utilized. **PLS-**SEM was selected over covariance-based SEM (CB-SEM) for the following methodologically justified reasons: (1) the study is prediction-oriented, aiming to explain variance in sustainable learning performance across a complex multi-construct model, which aligns with PLS-SEM's core strengths; (2) the model includes a large number of constructs and indicators, and PLS-SEM performs robustly under such conditions without imposing strict distributional assumptions; (3) the study incorporates moderation effects via interaction terms, for which PLS-SEM's two-stage approach provides a well-validated estimation procedure; and (4) the sample size of 623, while adequate for PLS-SEM, may pose convergence challenges for CB-SEM with models of this complexity. These considerations collectively justify PLS-SEM as the primary analytical approach (Hair et al., 2021). In terms of the order through which analysis was conducted, the following order was followed: (a) The measurement model was assessed to test the reliability and validity of all constructs. The reliability refers to Cronbach's alpha and composite reliability, while the validity includes convergent and discriminant validities. All indicators with inadequate loadings were examined and considered as part of the assessment only when their inclusion was found necessary based on their theoretical backing; (b) The structural model was assessed to evaluate the statistical significance of all proposed direct and indirect relationships and the moderator effects. This was done using bootstrapping techniques in which 5,000 bootstraps were conducted. Path coefficients, effect sizes (*f*^2^), determination of coefficient (*R*^2^), and predictive relevance (*Q*^2^) by means of blindfolding were determined as measures of explanation and prediction. The Standardized Root Mean Square Residual (SRMR) was determined as a measure of overall goodness of fit. It is considered acceptable if below 0.08. PLS prediction technique was also performed to assess the out-of-sample predictive performance of the structural model (Henseler and Chin, 2010).

Qualitative phase was carried out after completion of quantitative phase, as was planned under the explanatory sequential research approach. The interviews were transcribed verbatim, thematically analyzed via the six-phase approach outlined in Section 4.2, and used for generation of explanations of the processes and contexts that lie behind quantitative structural relationships. Integration of both qualitative and quantitative data proceeded through application of the formal mixed-methods research integration strategy involving three steps: (1) triangulation, where qualitative themes were systematically examined against quantitative path findings in order to check on convergence, divergence or contradiction between the two approaches; (2) joint display analysis, where quantitative findings and their qualitative counterparts were presented; and (3) meta-inference, where the results obtained in both phases were synthesized in order to draw general conclusions that would be beyond reach for either of the two phases separately. Such an approach guarantees that the results of qualitative analysis become a true analytical input and not just illustrative confirmation of statistical findings.

## 5 . Results and discussion

### 5.1 Initial data analysis

Table 2 provides an overview of the descriptive statistics and indicators of normality for each of the constructs used in this study. Results showed that study participants demonstrated moderate levels of artificial intelligence (AI) literacy, as evidenced by mean scores between 2.95 and 3.04, which indicates a reasonable comprehension of multiple aspects of artificial intelligence: principles, capabilities, limitations, and ethical implication. Study participants' scores exhibited a standard deviation of 1.25–1.30, along with skewness and kurtosis values close to zero, indicating that the responses of participants were reasonably distributed around the mean and that there was no significant multicollinearity at the item level, consistent with the item-level VIF values reported in Table 2. Construct-level full collinearity VIF values are reported and discussed separately in Section 4.1 (Hair et al., 2021). Results from SRL on average reflected similar means (2.98-3.09) and variances that would indicate a moderate variability of study participants' abilities to plan, monitor, and regulate their learning when using AI tools. Results from the evaluation of critical information generated by AI indicated that the use of AI provided sufficient means for verifying output and provided sufficient means for determining the validity of the sources used in these outputs (M = 2.99–3.01). In addition, this set of measures showed a low to non-existence of multicollinearity (as also indicated by low VIF), and skewness and kurtosis values close to zero, further supporting the validity and reliability of the surveys used to estimate this information.

**Table 2:** Descriptive analysis and data normality.

Name	Items	Mean	Standard deviation	Excess kurtosis	Skewness	VIF
AI literacy	I understand the basic principles of how generative AI systems work	2.981	1.301	−1.094	0.040	4.261
	I am aware of the capabilities of generative AI tools such as ChatGPT or Copilot	3.000	1.292	−1.066	0.031	3.289
	I understand the limitations and potential errors of AI-generated outputs	2.984	1.303	−1.089	−0.014	2.979
	I know how to evaluate whether AI-generated information is accurate	2.950	1.295	−1.073	0.026	3.527
	I am familiar with ethical issues related to the use of AI technologies	3.024	1.253	−0.965	−0.011	2.883
	I understand how AI technologies can be applied in academic or professional tasks	2.994	1.272	−0.982	0.021	2.607
	I understand the basic principles of how generative AI systems work	3.042	1.089	−0.625	−0.008	4.261
Self-regulated learning	I plan how to use AI tools to support my learning	2.995	1.062	−0.536	−0.023	2.215
	I choose appropriate strategies when using AI for academic tasks	2.998	1.053	−0.527	0.069	2.029
	I monitor my understanding when using AI tools	2.995	1.041	−0.380	0.018	1.815
	I check whether I am learning effectively while using AI	2.998	1.073	−0.614	−0.028	2.74
	I stay focused on my learning goals when using AI	3.018	1.036	−0.574	−0.044	2.264
	I adjust my learning strategies when AI does not meet my needs	2.982	1.093	−0.618	0.094	2.819
	I change how I use AI if it does not help me understand the material	3.018	1.038	−0.470	−0.035	2.014
	I try different ways of using AI to improve my learning	3.056	1.032	−0.492	−0.060	2.957
	I reflect on how effectively I use AI for my learning	3.026	1.078	−0.655	0.026	1.866
	I evaluate whether AI has improved my learning outcomes	3.087	1.080	−0.591	−0.081	2.058
Critical AI evaluation	I check the sources of AI-generated content before relying on it	3.008	1.118	−0.694	0.012	1.866
	I verify AI responses using other sources or references	2.989	1.095	−0.618	0.059	3.052
	I evaluate whether AI-generated information is reliable	3.000	1.120	−0.708	0.096	2.732
	I check the accuracy of AI-generated content before using it	3.010	1.093	−0.619	0.025	2.999
	I critically assess AI outputs to ensure they are correct and meaningful	3.005	1.068	−0.612	0.070	2.643
Effective AI use	I use AI to assist in collecting and organizing information for my academic tasks	3.013	1.179	−0.853	0.005	2.652
	I use AI to help me present information clearly (e.g., summaries or structured outputs)	3.019	1.224	−0.977	−0.005	3.179
	I use AI to support problem-solving in my coursework	3.016	1.196	−0.850	−0.031	2.863
	I use AI to generate initial ideas or outlines for my assignments	2.944	1.213	−0.921	0.021	2.537
	I use AI to improve the clarity and quality of my writing	3.003	1.169	−0.814	0.072	3.299
	I use AI to refine sentence structure and improve the logical flow of my work	2.997	1.194	−0.853	0.108	3.741
	I use AI to integrate knowledge from different subjects or perspectives	2.976	1.155	−0.815	0.047	3.132
	I use AI to expand my thinking and explore alternative solutions to problems	2.987	1.253	−0.983	0.073	3.247
	I use AI to support reflection on my learning	2.998	1.212	−0.888	0.025	3.445
AI over-reliance	I rely heavily on AI to complete my academic assignments	2.990	1.021	−0.567	0.028	1.638
	I find it difficult to perform academic tasks without AI support	2.994	0.978	−0.343	0.199	2.141
	I use AI so frequently that I depend on it for my academic work	3.039	0.964	−0.442	0.203	2.065
	I feel the need to use AI regularly to maintain my academic performance	3.021	1.005	−0.523	0.139	1.909
	I find it difficult to control how much I rely on AI for my studies	3.067	0.913	−0.037	0.171	2.203
Polychronicity	I like to work on several academic tasks at the same time	3.059	1.289	−1.026	−0.034	4.48
	I prefer to complete one academic task at a time rather than working on multiple tasks simultaneously *(r)*	3.075	1.310	−1.083	−0.020	3.133
	I believe students should try to handle multiple academic activities at once	3.055	1.312	−1.094	−0.080	2.325
	I believe it is better to complete one academic task before starting another *(r)*	3.066	1.291	−1.045	−0.042	2.112
	I prefer being engaged in several academic tasks or assignments at the same time	3.066	1.327	−1.121	−0.088	3.683
Sustainable learning performance	I have gained new and useful knowledge that improves my learning	2.979	0.911	−0.166	−0.010	1.401
	My learning has improved because I apply new knowledge effectively	2.979	1.000	−0.497	−0.055	1.292
	I am able to retain and use what I learn over time	2.947	0.953	−0.275	0.072	1.649
	I can apply my learning independently to solve academic problems	2.937	0.947	−0.390	0.000	1.487
	I can apply what I learn in new and different situations	2.978	0.832	−0.180	0.076	1.564
	My learning has improved because I apply new knowledge effectively	3.006	0.837	−0.067	−0.012	4.48

Generalized uses for AI applications by students had moderate average scores (between 2.94 and 3.02 with adequate range/distribution) suggesting that they effectively utilize these tools for processing information, solving problems, and reflecting upon information. They showed moderate levels of dependency on AI and moderate levels of variation (2.99–3.07). The results illustrated that while students do use AI to some degree, they are not overly reliant upon it. Additionally, the data from the polychronicity measures demonstrate that students exhibit moderate levels of propensity to multitask synchronously (M 3.06–3.08) and demonstrate adequate levels of variation, therefore allowing for the use of moderation in this area of study. The descriptive of sustainable learning performance showed slightly less variation (2.94–3.01); however, the lower AVE associated with this construct may be a result of one item having a higher VIF (4.48). However, the measures of skewness and kurtosis indicate a normal distribution. Overall, the results presented in Table 2 indicate that the data analyzed for this study were normally distributed and satisfactory in terms of multicollinearity and were suitable for structural equation modeling and testing of hypotheses.

### 5.2 Total variance explained

The research study presented in this paper utilized the Total Variance Explained method for the purpose of assessing the variability in relation to a given study, and the contribution of each variable to total Variance explained by the study. A total variance cumulative figure of 87.153% was produced as a result of this type of analysis as shown in Table 3, which is much greater than the usual, recommended minimum of 50%. The eigenvalue criterion was also met by the analysis (2 eigenvalues >1.0), thus confirming the retention of the 2 components for the data being analyzed. This redistribution of variance shown in the rotated factor solution does not affect the total cumulative variance but rather supports a stable and well-defined structure. Based on these findings, the researchers have concluded that the data exhibit sufficient reliability and appropriateness to allow for further multivariate analysis and therefore, the potential use of SEM-based methods (Hair et al., 2021).

**Table 3:** Total variance explained.

Component	Initial eigenvalues			Extraction sums of squared loadings			Rotation sums of squared loadings		
	Total	% of variance	Cumulative %	Total	% of variance	Cumulative %	Total	% of Variance	Cumulative %
1	4.666	66.658	66.658	4.666	66.658	66.658	4.611	65.869	65.869
2	1.435	20.495	87.153	1.435	20.495	87.153	1.490	21.284	87.153
3	0.293	4.190	91.343						
4	0.259	3.698	95.042						
5	0.154	2.203	97.245						
6	0.113	1.621	98.866						
7	0.079	1.134	100.000						

### 5.3 Measurement model assessment

**Figure 2:**
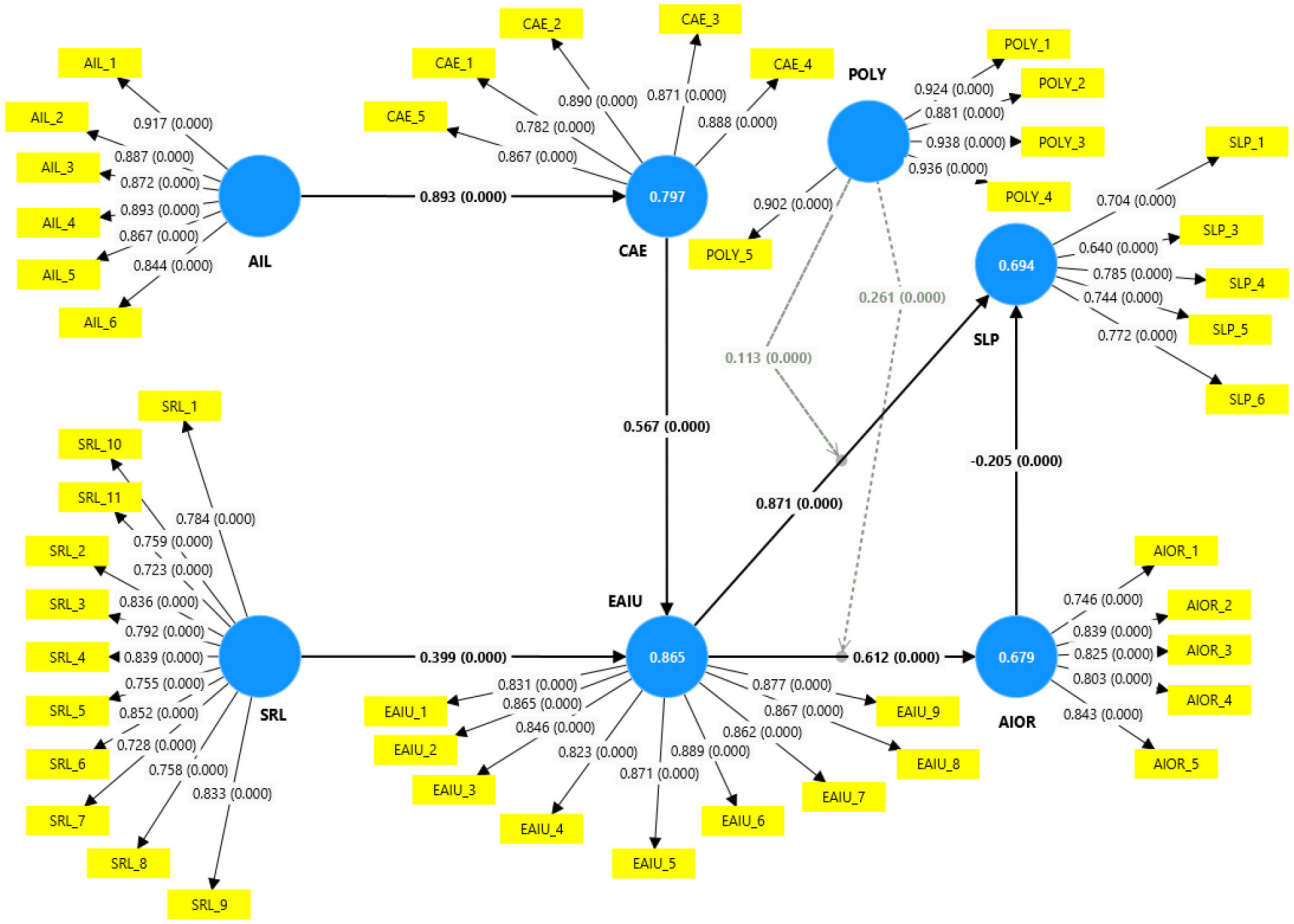
Structural model.

Results for the assessment of the measurement model are presented in Table 4 and Figure 2. The measurement model includes Factor Loading (FL), Cronbach's Alpha (α), Composite Reliability (CR), and Average Variance Extracted (AVE). The constructs showed adequate convergent validity (CR) and reliability (α) to continue with structural analysis. The construct of Self-Regulated Learning (SRL) had strong internal consistency with a Cronbach's Alpha (α) of 0.942, a Composite Reliability (CR) of 0.954, an FL of 0.723–0.852, and an Average Variance Extracted (AVE) of 0.622. Therefore, items that measure SRL are reliable measurements of the SRL behavior of students who are using AI to facilitate their learning, and thus, these measurement items should reliably reflect the SRL behaviors such as planning, monitoring, and self-regulation, of students using AI to support their studies.

**Table 4:** Measurement model.

Construct	Indicator	FL	α	Cr	AVE
Self-regulated learning	AIL_1	0.917	0.942	0.954	0.775
	AIL_2	0.887			
	AIL_3	0.872			
	AIL_4	0.893			
	AIL_5	0.867			
	AIL_6	0.844			
AI over-reliance	AIOR_1	0.746	0.870	0.906	0.606
	AIOR_2	0.839			
	AIOR_3	0.825			
	AIOR_4	0.803			
	AIOR_5	0.843			
Critical AI evaluation	CAE_1	0.782	0.912	0.934	0.712
	CAE_2	0.890			
	CAE_3	0.871			
	CAE_4	0.888			
	CAE_5	0.867			
Effective AI use	EAIU_1	0.831	0.956	0.962	0.738
	EAIU_2	0.865			
	EAIU_3	0.846			
	EAIU_4	0.823			
	EAIU_5	0.871			
	EAIU_6	0.889			
	EAIU_7	0.862			
	EAIU_8	0.867			
	EAIU_9	0.877			
Polychronicity	POLY_1	0.924	0.952	0.963	0.840
	POLY_2	0.881			
	POLY_3	0.938			
	POLY_4	0.936			
	POLY_5	0.902			
Sustainable learning performance	SLP_1	0.704	0.781	0.851	0.534
	SLP_3	0.640			
	SLP_4	0.785			
	SLP_5	0.744			
	SLP_6	0.772			
Self-regulated learning	SRL_1	0.784	0.939	0.947	0.622
	SRL_10	0.759			
	SRL_11	0.723			
	SRL_2	0.836			
	SRL_3	0.792			
	SRL_4	0.839			
	SRL_5	0.755			
	SRL_6	0.852			
	SRL_7	0.728			
	SRL_8	0.758			
	SRL_9	0.833			

The reliability of AI Over-Reliance was also shown (α = 0.870; CR = 0.906), as well as loadings ranging from 0.746 to 0.843, and AVE equal to 0.606, indicating that the items consistently measure students' dependency on AI for academic purposes (Risko and Gilbert, 2016; Zhang et al., 2024). Critical AI Evaluation had a strong loading (0.782–0.890), an excellent level of reliability (α = 0.912; CR = 0.934), and an AVE equal to 0.712, confirming that the scale adequately reflects students' capacity to appraise and critically evaluate AI-generated outputs. Effective AI Use had an extremely high degree of reliability (α = 0.956; CR = 0.962), along with loadings ranging from 0.823 to 0.889 and an AVE equal to 0.738, indicating that the construct successfully captures students' productive use of AI tools for learning activities (Chiu et al., 2023; Tsai and Chai, 2012). Similarly, Polychronicity had an excellent level of reliability (α = 0.952; CR = 0.963), with loadings ranging from 0.881 to 0.938, and an AVE equal to 0.840. Finally, Sustainable Learning Performance demonstrated adequate loadings (0.640–0.785), and reliability (α = 0.781; CR = 0.851) but a slightly lower AVE (0.534). This means that while the items were able to capture students' retention, application, and independent learning, there was still some variance that could not be fully accounted for, and this variance is consistent with earlier studies using the same measures of performance (Holmes et al., 2022; Zhang et al., 2024). As shown in Table 3, all constructs meet the recommended threshold of reliability (α > 0.7; CR > 0.7) and convergent validity (AVE > 0.5), therefore validating the measurement model for subsequent structural equation modeling (Hair et al., 2021).

The results of the Heterotrait-Monotrait Ratio (HTMT) analysis, used to assess discriminant validity for the constructs included in this study, are provided in Table 5. Constructs that have HTMT values of less than 0.85 are considered to be adequate in providing discriminant validity and demonstrating that each construct provides a unique perspective on the model (Henseler et al., 2015). As can be seen in Table 5, the HTMT values for AI Literacy, AI Over-Reliance, Critical AI Evaluation, Effective AI Use, Polychronicity, Sustainable Learning Performance and Self-Regulated Learning range from 0.030 to 0.859. The greatest HTMT ratio was found between Effective AI Use and Sustainable Learning Performance (HTMT = 0.859), which, while approaching the conservative 0.85 threshold, remains below the widely accepted 0.90 cutoff identified by (Kline, 2023). In other words, even though constructs are unique, there can be overlapping elements among them, especially in relation to Effective AI Use and Sustainable Learning Performance. Indeed, the existence of this overlapping is theoretically expected since effective AI use is considered to be the proximal antecedent of sustainable learning performance in the developed model, meaning that these constructs are supposed to show strong relations to each other. Yet, despite being theoretically connected, these concepts are unique from an operational standpoint, with Effective AI Use referring to the utilization of AI by students while completing their learning assignments (problem-solving, reflections, etc.), and Sustainable Learning Performance being used to measure its long-lasting outcomes. This difference is underlined by the fact that the obtained HTMT value of 0.859 is above 0.90 but not equal to it, as well as the theoretical model that suggests considering EAIU as an SLP's predictor. The HTMT Ratio shows that Polychronicity exhibits very low HTMT values (i.e., 0.030 to 0.495), which suggests that it serves as a moderated variable and is conceptually distinct from the primary predictors and outcomes. Additionally, although AI Over-Reliance has a moderate HTMT; values with respect to AI Literacy (0.665), Critical AI Evaluation (0.682), and Effective AI Use (0.702), its similarities with these three constructs support the theoretical separation of AI Over-Reliance but also reflects expected behavioral relationships between them. Lastly, Sustainable Learning Performance displays high HTMT Values with Effective AI Use (0.859), Critical AI Evaluation (0.813) and AI Literacy (0.800), which is supported by the theoretical model; thus, these Constructs are expected to contribute to learning outcomes. Finally, the data presented in Table 4 supports that all Constructs within the Proposed Model meet the necessary Discriminant Validity criteria and therefore, can be utilized in further structural analysis within PLS-SEM.

**Table 5:** Discriminant validity.

Construct	1	2	3	4	5	6	7
1. AI literacy							
2. AI over-reliance	0.665						
3. Critical AI evaluation	0.761	0.682					
4. Effective AI use	0.749	0.702	0.769				
5. Polychronicity	0.030	0.495	0.040	0.030			
6. Sustainable learning performance	0.800	0.334	0.813	0.859	0.389		
7. Self-regulated learning	0.737	0.686	0.715	0.728	0.043	0.767	

### 5.4 Hypotheses testing

Table 6 and Figure 2, presents the results of the structural model assessment using a bootstrapping procedure with 5,000 resamples. The findings indicate that all hypothesized relationships are statistically significant, providing strong support for the proposed model.

**Table 6:** Hypotheses results.

Path	β	Standard deviation	T statistics	*P* values	*f* ^2^
AIL → CAE	0.893	0.008	111.167	0.000	3.916
AIOR → SLP	−0.205	0.039	5.291	0.000	0.044
CAE → EAIU	0.567	0.028	20.081	0.000	0.671
EAIU → AIOR	0.612	0.024	25.574	0.000	1.160
EAIU → SLP	0.871	0.029	30.115	0.000	1.139
SRL → EAIU	0.399	0.028	14.015	0.000	0.332
POLY × EAIU → AIOR	0.261	0.025	10.422	0.000	0.227
POLY × EAIU → SLP	0.113	0.025	4.452	0.000	0.036
		AIOR	CAE	EAIU	SLP
*R* ^2^		0.679	0.797	0.865	0.694
*Q* ^2^		0.438	0.578	0.626	0.360

H1 is supported: AI Literacy is significantly and positively associated with Critical AI Evaluation (β = 0.893, *t* = 111.167, *p* < 0.001, *f*^2^ = 3.916), with a very large effect size. This finding suggests that students with greater understanding of AI capabilities and limitations engage more actively in verifying and critically assessing AI outputs, consistent with the AI Literacy Framework and recent empirical work on critical AI engagement in higher education (Bewersdorff et al., 2025). H2 is supported: Critical AI Evaluation is positively associated with Effective AI Use (β = 0.567, *t* = 20.081, *p* < 0.001, *f*^2^ = 0.671), with a large effect size. This finding suggests that students who habitually verify AI outputs are more likely to engage with AI tools purposefully and productively—consistent with research on critical thinking in AI-assisted learning (Oates and Johnson, 2025; Melisa et al., 2025). H3 is supported: Self-Regulated Learning is positively associated with Effective AI Use (β = 0.399, *t* = 14.015, *p* < 0.001, *f*^2^ = 0.332), with a medium-to-large effect size. This finding suggests that students who plan, monitor, and adapt their learning strategies engage with AI tools more purposefully, consistent with SRL Theory and recent research on metacognitive AI engagement (Xu et al., 2025; Zhang et al., 2025). H4 is supported: Effective AI Use is positively associated with Sustainable Learning Performance (β = 0.871, *t* = 30.115, *p* < 0.001, *f*^2^ = 1.139), with a very large effect size. This finding suggests that students who engage with AI as an active cognitive partner demonstrate stronger perceived knowledge retention, independent application, and transfer, consistent with research on AI-assisted deep learning (Chen and Chang, 2024; Yang, 2025). H5 is supported and represents the central paradox of the model: Effective AI Use is positively associated with AI Over-Reliance (β = 0.612, *t* = 25.574, *p* < 0.001, *f*^2^ = 1.160), with a very large effect size. This counterintuitive finding suggests that students who engage most productively with AI are simultaneously most at risk of habitual dependency, consistent with the cognitive habituation mechanism theorized in Section 3.5 and with recent evidence on LLM-enabled AI dependency in education (Zhai et al., 2024). H6 is supported: AI Over-Reliance is negatively associated with Sustainable Learning Performance (β = −0.205, *t* = 5.291, *p* < 0.001, *f*^2^ = 0.044). While the effect size is small, the statistical significance and theoretical importance are substantial: it documents that habitual AI dependency is associated with reduced independent knowledge retention, application, and transfer, the core components of sustainable learning performance as operationalised in this study, consistent with Cognitive Offloading Theory and recent research on AI-induced skill atrophy (Zhai et al., 2024; Karamuk, 2025). H7b is supported: Polychronicity significantly moderates the Effective AI Use, AI Over-Reliance relationship (β = 0.261, *t* = 10.422, *p* < 0.001, *f*^2^ = 0.227), with a medium effect size. Students with higher multitasking tendencies are more susceptible to AI dependency as effective use increases, consistent with the individual-difference moderator role theorized in Section 3.7. H7a is also supported: Polychronicity moderates the Effective AI Use, Sustainable Learning Performance relationship (β = 0.113, *t* = 4.452, *p* < 0.001, *f*^2^ = 0.036). While the effect size is small, the finding is statistically significant and theoretically important: the learning benefits of effective AI use are attenuated for students with higher multitasking tendencies, who experience greater cognitive fragmentation during AI-assisted study. Furthermore, the ability of this model to explain the existing variance of total Critical AI Evaluation (*R*^2^ = 79.7%), Effective AI Use (*R*^2^ = 86.5%), AI Over-Reliance (*R*^2^ = 67.9%), and Sustainable Learning Performance (*R*^2^ = 69.4%) indicates an overall strong explanatory capability . All predictive relevance (*Q*^2^) values within the model were above 0.000 (between 0.360 and 0.626), confirming this model's high predictive power.

**Figure 3:**
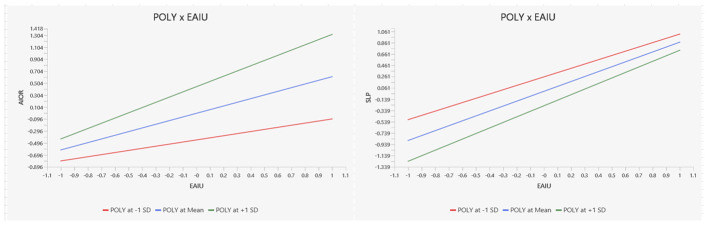
Moderation of polychronicity.

The moderation graphs in Figure 3, illustrate the conditional effects of polychronicity on the relationships between effective AI use and the two outcome variables.

One notable result of the study is that the slopes of all three levels of polychronicity in the first relationship (effective AI use and AI over-reliance) were positive, suggesting that higher levels of effective AI use lead to higher levels of over-reliance on AI. This also implies that the higher the level of polychronicity, the more pronounced the effect of effective AI use on AI over-reliance. In addition, there was a tendency for higher polychronicity to have a stronger effect on AI over-reliance for those whose use of AI increased than for those whose use of AI decreased. Thus, the relationship between effective AI use and AI over-reliance is moderated (or influenced) by the level of polychronicity.

All slopes in the second interaction are also positive, therefore confirming that the use of effective AI is beneficial to improving learning performance from a sustainability standpoint for all polychronicity levels. The slope is steepest at low polychronicity (−1 SD), then becomes flatter as polychronicity increases (+1 SD). This indicates that effective AI use produces a more substantial effect on sustainable learning performance at lower levels of polychronicity than at higher polychronicity levels. Thus, individuals who possess a lower degree of polychronicity experience a larger return when using effective AI for sustainable learning outcomes than do individuals who possess a higher degree of polychronicity.

### 5.5 Qualitative results

The qualitative analysis revealed several key themes that explain how generative AI influences students' learning processes and outcomes. The findings are organized into five main themes: (1) AI literacy and critical evaluation, (2) effective AI use for learning, (3) AI over-reliance, (4) role of self-regulated learning, and (5) multitasking behavior (polychronicity).

#### 5.5.1 Theme 1: AI Literacy Enhances Critical Evaluation

Educators consistently emphasized that students will have an enhanced capability to analyze, evaluate, and critique what the AI produces. When students are able to identify the limitations of an AI, they will tend to question the responses given by the AI, validate the information, and avoid relying on AI-generated content entirely. One participant noted:

“*Students who understand how AI works are more cautious. They don't just copy answers, they check and verify them.”*

Another educator highlighted:

“*AI literacy makes a big difference. Without it, students tend to trust AI too much, even when the information is incorrect.”*

These findings support the quantitative results by showing that AI literacy strengthens students' ability to engage in critical evaluation, which is essential for effective learning.

#### 5.5.2 Theme 2: Effective AI Use Improves Learning Outcomes

According to participants, proper utilization of AI tools can greatly enhance the learning experience of students; when used properly, AI tools enhance students' ability to learn through improving their conceptual understanding, organizing their thoughts into coherent structures, and increasing their academic productivity.

An educator explained:

“*Students use AI to clarify difficult concepts and generate ideas. It really helps them understand topics faster.”*

Another stated:

“*AI is very useful when students use it as a support tool rather than a replacement for thinking.”*

However, participants believed that these advantages to the use of AI tools are contingent upon the strategic use of students using AI tools; this is also consistent with what was established in the quantitative aspect of this study regarding the use of effective AI tools.

#### 5.5.3 Theme 3: AI Over-Reliance Undermines Learning

Despite its benefits, many educators expressed concerns about students becoming overly dependent on AI tools. Over-reliance was described as a major issue that reduces independent thinking and problem-solving skills.

One participant remarked:

“*Some students rely too much on AI. They don't try to solve problems on their own anymore.”*

Another noted:

“*When students depend heavily on AI, their critical thinking and creativity decline.”*

These insights strongly support the negative relationship between AI over-reliance and sustainable learning performance observed in the quantitative findings.

#### 5.5.4 Theme 4: Role of Self-Regulated Learning

According to the emphasis placed by educators on the importance of self-regulation with regard to the effects (whether good or bad) of AI use, those students who demonstrate a greater ability to regulate themselves will leverage the benefits of artificial intelligence and be less likely to rely too heavily on it.

As one educator explained:

“*Students who plan and monitor their learning use AI more effectively. They know when to rely on it and when to think independently.”*

Another added:

“*Self-discipline is key. Without it, students can easily misuse AI tools.”*

These findings reinforce the importance of self-regulated learning in shaping effective AI use and learning outcomes.

#### 5.5.5 Theme 5: Polychronicity and Multitasking Effects

The role of polychronicity emerged as an important factor influencing how students interact with AI tools. Educators observed that students who frequently multitask are more likely to rely on AI as a shortcut to manage multiple tasks simultaneously.

One participant stated:

“*Students who handle many tasks at once tend to use AI to save time, but they don't always learn deeply.”*

Another explained:

“*Multitasking students often depend on AI to complete tasks quickly, which can lead to shallow learning.”*

These findings provide qualitative support for the moderating effects observed in the quantitative analysis, particularly the strengthening of AI over-reliance among highly polychronic students.

### 5.6 Integration of quantitative and qualitative findings

The formal mixed-method integration strategy, comprising triangulation, joint display analysis (Table 7), and meta-inference, enables conclusions that transcend what either phase could produce independently. High convergence between quantitative structural findings and qualitative thematic evidence across all pathways strengthens confidence in the structural model. A key meta-inference is that the impact of generative AI on learning is fundamentally contingent on student agency: not AI access or use frequency *per se*, but the quality and intentionality of engagement. Students who bring AI literacy, self-regulation, and critical evaluation to their interactions achieve sustainable learning gains; those who engage passively risk consolidating dependency. Educators noted that students with higher AI literacy were more likely to' abilities to adequately (critically) evaluate the quality and usability of AI-based outputs. Through qualitative analysis, educators indicated that students who understand the workings of AI are much more apt to validate, research or critically assess AI-generated information. This indicates that in addition to improving a learner's technical know-how of AI, the enhanced cognitive skills cultivated by AI literacy allow learners to think analytically and reflectively when utilizing AI-supported learning. Furthermore, qualitative findings reinforce the quantitative finding of a positive impact from Critical AI Evaluation and Self-Regulated Learning on Effective Use of AI. Educators indicated that learners who actively engage in monitoring their learning and verifying AI-generated outputs tended to employ AI-based tools in a more intentional manner, resulting in improved academic performance. This supports the important complementarity of Cognitive Evaluation and Self-Regulation in determining both successful use of and engagement with AI-based tools.

**Table 7:** Joint display of quantitative and qualitative findings.

Hypothesis	Quantitative result	Qualitative theme	Interpretation
H1: AIL → CAE	Significant (+)	AI literacy improves evaluation	Students with AI knowledge verify outputs
H2: CAE → EAIU	Significant (+)	Critical thinking enhances usage	Evaluation leads to better AI use
H3: SRL → EAIU	Significant (+)	Role of self-regulation	Planning & monitoring improve AI use
H4: EAIU → SLP	Significant (+)	AI improves learning	AI supports understanding & performance
H5: EAIU → AIOR	Significant (+)	Dependency risk	Frequent use leads to reliance
H6: AIOR → SLP	Significant (–)	Negative learning impact	Over-reliance reduces thinking
H7: Moderation (POLY → AIOR)	Significant	Multitasking effect	Multitasking increases dependence
H8: Moderation (POLY → SLP)	Significant	Reduced focus	Multitasking weakens deep learning

Taken together, the integrated findings support a theoretically coherent and empirically robust account of how generative AI shapes sustainable learning in higher education. The dual-pathway model—in which the same behavior (effective AI use) simultaneously promotes learning and risks dependency, with individual differences (polychronicity) determining the balance—provides a nuanced alternative to both uncritical enthusiasm for AI in education and blanket concern about its risks. The evidence suggests that AI is neither inherently beneficial nor harmful: its impact depends on the cognitive strategies, metacognitive skills, and attentional capacities that students bring to their AI interactions. This conclusion has significant implications for educational theory, AI literacy programme design, and institutional policy on generative AI in higher education.

### 5.7 Discussion

Using an explanatory sequential mixed methods approach, this study investigated the joint effects of generative AI on sustainable learning performance among Chinese university students by using the PLS-SEM analysis results complemented by qualitative views from educators. This discussion places the results of the study into context by looking at the existing literature about generative AI, its usage in education, engagement with metacognitive AI, cognitive dependence, as well as collaborative human-AI learning to provide insight into the study's capacity for learning in an environmentally sustainable manner and use of quantitative and qualitative results to gain insights into positive and negative impacts of generative AI on student learning performance. By combining the quantitative statistical results obtained through multiple educators with the qualitative views and insights from qualitative research, the reliability of the results is enhanced.

First, the highly significant positive relationship between AI literacy and critical evaluation of AI (β = 0.893, *f*^2^ = 3.916) builds upon the digital literacy literature by proving how AI literacy can impact evaluative actions performed toward specific outputs created by the use of the generative, probabilistic LLMs -a uniquely different challenge which cannot be fully covered by generic digital literacy approaches (Oates and Johnson, 2025). It corroborates with the proposed AI Literacy Framework as well as with recent findings in the area of critical AI engagement among higher education students (Bewersdorff et al., 2025). This finding also aligns with prior research (Georgopoulou et al., 2025), that educators believe that the better understanding a student has of AI systems, the more they are likely to question, verify and critically analyze the outputs of AI systems. In addition, the data from our study found that AI literacy encompasses more than just technical knowledge; it includes cognitive awareness, which enables students to engage with AI-supported learning environments in an analytical manner. Secondly, the data from our study indicate that critical evaluation of AI and self-regulation of students' learning work together to produce effective use of AI. The quantitative data from our study indicate that there are significant positive relationships, consistent with (Nkomo et al., 2021), and the qualitative data from our study find that students who actively monitor their learning and evaluate AI outputs tend to use AI tools with more strategy. This means that effective use of AI is determined by both cognitive evaluation and metacognitive regulation. The findings of this study provide theoretical support for the premise of self-regulation in learning, where learners have control over and adapt their learning processes to produce optimal learning outcomes.

Third, the large positive association between effective AI use and sustainable learning performance (β = 0.871) confirms that strategic AI engagement supports knowledge retention, independent application, and cross-contextual transfer, consistent with recent research on human-AI collaborative learning demonstrating that AI tools function most effectively when they augment rather than replace student cognitive effort (Yang, 2025). Educators corroborated this, noting that students who use AI to clarify concepts and generate reflective feedback exhibit deeper engagement. Critically, this research provides evidence of the key paradox: successful usage of AI serves as a powerful determinant of AI overdependence (β = 0.612, *f*^2^ = 1.160). This paradoxical two-stage pathway constitutes the major theoretical contribution made by this research. It supports the cognitive habituation theory proposed in Chapter 3.5. The more students are experiencing efficiency provided by AI, the higher the cost associated with independent engagement becomes, leading students to make AI use their default tool. Contemporary research on the application of AI in education has suggested such risk related to LLM-powered instruction, suggesting that the convenience of AI-generated results can serve as a ground for escalation of cognitive offloading process (Zhai et al., 2024). Qualitative analysis revealed the context: teachers reported that the students who used AI most often were often least willing to engage independently. Chen and Chang (2024) demonstrate that AI can provide cognitive support to the learner, allowing for a greater depth of understanding and longer-lasting effects on learning. The results also show an interesting phenomenon. There are certain advantages from using artificial intelligence, but they depend on the manner in which this process takes place. If the student uses artificial intelligence properly, he or she gains knowledge; however, when used improperly, students do not get any benefit out of the AI use. It can be seen from the qualitative results obtained in the current research that there is dual impact from the use of artificial intelligence since, on one hand, students can benefit from its application, while on the other hand, there may appear some drawbacks as well. Using the AI correctly, students become better in terms of learning capacity. At the same time, they become more dependent on artificial intelligence as well. The educators showed some concerns about the increasing dependency on AI because, as students start using it more often, they might develop dependency on AI itself. These results are compatible with the theory of cognitive offloading, where reliance on external tools for cognitive labor leads to decreasing internal cognitive effort.

Fourth, the negative relation between AI over-reliance and sustainable learning performance (β = −0.205) shows practical significance, despite being smaller in size than the positive H4 path. This indicates that the tendency to use artificial intelligence tools in a habitual way has a strong connection to the inability of learners to independently accumulate and apply knowledge that is sustainable learning. In this context, findings from two systematic literature reviews support the theoretical relevance of the negative relationship between excessive use of technology and sustainable learning: studies have demonstrated a negative influence of excessive AI use on learners‘ critical thinking and autonomous problem-solving skills (Zhai et al., 2024; Karamuk, 2025). Despite the small size, this finding should not be overlooked because the negative pathway works partly as a balancing one in contrast to the positive H4 relationship. Namely, the positive influence of AI use on sustainable learning performance partially gets offset by the negative one, which implies that the overall impact depends on both factors. Thus, students who effectively use AI but do not form a tendency to rely on it demonstrate the best results in sustainable learning; however, dependency reduces their performance somewhat. Fifth, the moderating effect of polychronicity serves as an individual difference boundary condition on both paths. The discovery of polychronicity as a moderator of the effective-use to over-reliance (β = 0.261, *f*^2^ = 0.227) and of effective-use to SLP relationships (β = 0.113) resonates with literature on multitasking and fragmented cognition (Grawitch and Barber, 2013; Glass and Kang, 2019) and expands it by considering the use of AI. The educators' perception of multitasking learners using AI more often as a time management tool than as a means for learning supports the proposed mechanism: for polychronic individuals, engagement with generative AI would be instrumental rather than reflective, focused on task completion rather than understanding. This discovery implies important practical considerations concerning the design of AI-enabled learning systems, especially for learners who have to deal with multiple tasks simultaneously, which characterizes higher education regardless of its cultural specificity, but does not specifically apply to China.

## 6 Implications

There are many important contributions made by this research to the area of educational AI technology, integrated learning science and educational psychology. The first contribution is that it demonstrates that through research, we have now expanded our understanding of AI Literacy as a specific construct. The first contribution is that the definition of AI Literacy now includes not only the evaluation of data, but also the evaluation of the predicted outcomes of all types of generative (probabilistic) LLM outputs. The use of the term ‘Evaluation' in a general sense rather than evaluating a single piece of Digital Content (e.g., LLM generated outputs) is a very critical point toward advancing our knowledge of AI. The introduction of the term Critical AI Evaluation has been established as a new metacognitive driver (critical construct) and should encourage educators, students and parents to evaluate LLM generated content based on the context and environment in which they were created. By using the term Critical AI Evaluation, it is now evident that AI literacy is not just about having a knowledge of technology but is also an essential component for responsible and beneficial interaction with generative AI tools. The second contribution of this research is the advancement of the Self-Regulated Learning (SRL) Theory and how it can be used to advance how students are supported in their use of AI. It has previously been understood that SRL functions as a metacognitive driver to support the effective use of AI, in the same way that SRL supported the traditional methods of learning. The study confirmed that in AI-supported learning environments, the quality of AI output depends directly on the quality of the query made by the student and on the student's evaluative engagement with the output(s). Therefore, this research reaffirms that an individual's autonomous decision-making is the key contributor in supporting or inhibiting the ability for AI tools to create sustainable learning outcomes.

Third, the study makes a novel theoretical contribution by introducing and empirically documenting the dual-pathway model, in which effective AI use simultaneously activates a positive pathway to sustainable learning performance and a risk pathway to AI over-reliance. This advances Cognitive Offloading Theory beyond its traditional focus on the negative effects of external tool reliance, demonstrating that offloading is conditionally beneficial: it supports learning when governed by AI literacy and SRL, but generates habitual dependency when those regulatory mechanisms are insufficient. The paradox that highly effective AI users are simultaneously most at risk of dependency is counterintuitive and has not been empirically established in prior LLM-based education research. Fourth, the study advances understanding of individual differences in AI-supported learning by demonstrating that polychronicity, a stable multitasking preference. moderates both pathways in theoretically coherent directions, positioning it as a boundary condition for AI-supported learning effectiveness that is not culturally specific but operates through cognitive fragmentation mechanisms applicable across educational contexts. Fifth, the mixed-method integration strategy, comprising triangulation, joint display analysis, and meta-inference, demonstrates how formal integration of quantitative structural findings and qualitative educator perspectives generates insights about AI dependency and student agency that neither method could produce independently, constituting a methodological contribution to mixed-method AI education research.

The results have many implications for universities, policy-makers, and educators. As previously noted, AI literacy enables critical evaluation of AI. Therefore, universities should create a structured AI literacy curriculum that goes beyond technical skills to help students develop their ability to evaluate LLM output critically (e.g., putting them in a position to understand how LLMs generate probabilistically, hallucinate, and are context-dependent). Universities should also take into consideration both their institutional/cultural context and the established AI literacy core competency frameworks that have been validated internationally when designing curricula for this area. The substantial role of SRL in facilitating the effective use of AI indicates the need for universities to incorporate metacognitive practices into the implementation of AI. This can be done by incorporating reflective learning, goal-setting, and self-monitoring exercises into AI-embedded courses. For example, students can be required to articulate how they evaluated their LLM output and which elements they verified independently prior to executing the task for the first time. The finding that effective AI use predicts over-dependence on this technology highlights the need for assessment types to include multiple strategies to protect against cognitive dependence. Assessment types that require students to employ independent reasoning and to justify their responses independently of AI will develop a practice effect on abilities that AI usage may compromise. The moderating role of polychronicity suggests that students with significant tendencies to multitask should be specifically addressed in AI-enabled contexts by practitioners who understand the risk of dependence via cognitive fragmentation. Implementing structured protocols for AI use and pedagogical strategies that promote focused attention will aid students at increased risk due to the challenges associated with cognitive fragmentation. Fifth, the study underscores that AI-enabled learning environments should be designed as sustainable learning ecosystems that prioritize the development of durable, transferable competencies, independent reasoning, critical evaluation, and self-directed learning, rather than merely as tools for academic task completion efficiency.

## 7 Conclusion

This study addressed the growing debate on whether generative AI enhances or undermines student learning—a question that prior research has largely failed to resolve because it focused on adoption and perceived usefulness rather than learning consequences, and rarely integrated individual differences and cognitive risk mechanisms into a unified framework. To address this gap, this study developed and empirically tested a dual-pathway model integrating AI Literacy, Self-Regulated Learning Theory, and Cognitive Offloading Theory, combining three-wave time-lagged survey data from 623 Chinese university students with qualitative educator interviews analyzed through a formal mixed-method integration strategy. The findings demonstrate that higher AI literacy is associated with stronger critical AI evaluation, which combined with self-regulated learning, is associated with more effective and purposeful AI engagement. Effective AI use is in turn positively associated with sustainable learning performance—operationalised in this study as perceived knowledge retention, independent application, and cross-contextual transfer. However, the study also documents the central paradox: effective AI use is simultaneously and strongly associated with AI over-reliance, which negatively affects sustainable learning performance. Polychronicity moderates both pathways, such that students with higher multitasking tendencies show a stronger effective-use-to-over-reliance association and a weaker effective-use-to-SLP association. The qualitative findings provide explanatory depth by confirming and contextualizing the structural relationships and generating meta-inferences about the role of student agency and the invisibility of AI dependency to students themselves. Together, these findings demonstrate that the impact of generative AI on learning is contingent on how students engage with the technology, with significant implications for AI literacy education, metacognitive pedagogy, and the design of sustainable AI-enabled learning environments.

Despite its contributions, this study has several limitations that should be considered when interpreting the findings. First, Chinese universities were the sole source for the sampled participants; thus, the findings may not transfer across cultures and educational systems. For example, culturally related issues such as collectivist values, high workload expectations in school, and institutional acceptance of AI as tools may be factors that influence how students in China use generative AI differently than do students using generative AI in other cultures such as the U.S. or Europe. Further studies need to replicate this study with a geographic spread, across different educational systems, to determine if education systems can transfer the findings and to identify culturally based moderating factors to the identified relationships. Second, although the study incorporated a time-lagged design to reduce common-method bias and strengthen temporal inference, the study was ultimately cross-sectional, non-experimental, and lacks the strength of temporal separation establishing causality. For example, a student may have already been predisposed to using AI prior to engaging in an AI evaluation process and, therefore, may have engaged in the evaluation process differently than other students who were not predisposed. Further studies should consider incorporating experimental or longitudinal designs to better determine the direction and longevity of the identified relationships established through this research. Third, sustainable learning performance was assessed exclusively through self-report from the students. There were no objective measures of learning performance such as (1) grade-point average; (2) standardized methods of retention assessment; or (3) measures of observable behavior associated with retention of knowledge/skill. Although self-report scales are commonly used and many studies confirm their validity, the scales measure perceived learning outcomes, thus (a) future studies should also use objective performance measures to support the research's conclusion about potential actual long-term learning effects; and (b) as research continues, it will be interesting to monitor how students are using generative AI tools and compare with long-term retention of knowledge/skill and/or sustained performance. Lastly, all constructs were self-reported by the same sample using Likert type scales, suggesting an element of CMB. Although the use of time lagged measures, analysis of full collinearity through VIFs, and Harman's single factor tests was done in order to control for any such risk, CMB still cannot be totally avoided. It is hoped that future research could include multiple sources of data and markers of CMB.

## Data Availability

The datasets generated and/or analyzed during the current study are available from the corresponding author upon reasonable request.
